# Long-term outcome of a patient with Transcobalamin deficiency caused by the homozygous c.1115_1116delCA mutation in TCN2 gene: a case report

**DOI:** 10.1186/s13052-021-01007-6

**Published:** 2021-03-08

**Authors:** Francesco Martino, Alessandra Magenta, Maria Letizia Troccoli, Eliana Martino, Concetta Torromeo, Carolina Putotto, Francesco Barillà

**Affiliations:** 1 Department of Pediatrics Gynecology and Obstetrics, Sapienza University of Rome, Viale Regina Elena 324, 00161 Rome, Italy; 2 National Research Council of Italy (CNR), Institute of Translational Pharmacology IFT, Via Fosso del Cavaliere 100, 00133 Rome, Italy; 3 Clinical Analysis and Biochemistry Laboratory, Sant’Andrea Hospital, Via di Grottarossa, 1035/1039, 00189 Rome, Italy; 4 Department of Cardiovascular, Respiratory, Nephrology, Anesthesiology and Geriatric Sciences, Sapienza University of Rome, Viale del Policlinico 155, 00161 Rome, Italy

**Keywords:** Cobalamin, Deficiency, Transcobalamin, Megaloblastic anemia, Hydroxocobalamin

## Abstract

**Background:**

Transcobalamin deficiency is a rare autosomal recessive inborn error of cobalamin transport (prevalence: < 1/1000000) which clinically manifests in early infancy.

**Case presentation:**

We describe the case of a 31 years old woman who at the age of 30 days presented with the classical clinical and laboratory signs of an inborn error of vitamin B_12_ metabolism. Family history revealed a sister who died at the age of 3 months with a similar clinical syndrome and with pancytopenia. She was started on empirical intramuscular (IM) cobalamin supplements (injections of hydroxocobalamin 1 mg/day for 1 week and then 1 mg twice a week) and several transfusions of washed and concentrated red blood cells. With these treatments a clear improvement in symptoms was observed, with the disappearance of vomiting, diarrhea and normalization of the full blood count. At 8 years of age injections were stopped for about two and a half months causing the appearance of pancytopenia. IM hydroxocobalamin was then restarted sine die. The definitive diagnosis could only be established at 29 years of age when a genetic evaluation revealed the homozygous c.1115_1116delCA mutation of TCN2 gene (p.Q373GfsX38).

Currently she is healthy and she is taking 1 mg of IM hydroxocobalamin once a week.

**Conclusions:**

Our case report highlights that early detection of TC deficiency and early initiation of aggressive IM treatment is likely associated with disease control and an overall favorable outcome.

## Background

Vitamin B_12_ (B_12_) also known as cobalamin is absorbed at the terminal ileum level, associated with a glycoprotein of gastric origin (intrinsic factor), by means of an endocytosis mechanism, mediated by an enterocyte membrane receptor [1]. Subsequently the B_12_ is secreted in the bloodstream where it binds to the vector protein haptocorrin (HC, previously named transcobalamin I). In the enterocyte, the intrinsic factor - cobalamin complex dissociates and cobalamin reaches the portal circulation where it is bound to transcobalamin (formerly known as transcobalamin II [TC]) [1]. In this bound form it is recognized by the surface receptors of the user cells (mainly those tissues with proliferative activities such as those of the bone marrow and of the germinative layer of the epithelia) and introduced, through a process of micropinocytosis, inside the cell, where the B_12_ is freed from the vector by lysosomal acid hydrolases and reduced by cytoplasmic enzymes [2].

At this point the reduced B_12_ undergoes two separate metabolic processes to be converted into the two co-enzymatically active forms: in the cytosol, in the form of methyl-cobalamin, it becomes part of the catalytic process of the enzyme methionine synthase; in the mitochondrion undergoes an adenosylation process that forms adenosyl-cobalamin (coenzyme of methylmalonyl-CoA mutase).

Congenital errors of cobalamin metabolism can affect any stage of absorption, blood transport and intracellular use.

The only congenital absorption deficit is due to a genetic alteration of the enterocytic receptor (Imerslund-Grasbeck syndrome), which is characterized by low serum cobalamin levels, a normal intrinsic factor and megaloblastic anemia [3].

TC deficiency is a typical transport error characterized by megaloblastic anemia with normal serum levels of B_12_ [4]. In this form there is a combined deficiency of synthesis of both methylcobalamin (methionine synthase coenzyme) and adenosyl cobalamin (methylmalonyl-CoA mutase coenzyme), with the consequent possibility of homocystinuria and methylmalonic aciduria. A massive homocystinuria with methylmalonic aciduria is characteristic of the combined deficits of the common intracellular metabolism of the B_12_ [5].

Isolated deficiencies of the specific metabolic pathways of adenosylcobalamin and methylcobalamin lead, respectively, to methylmalonic aciduria and homocystinuria. In the first case there will be the forms of methylmalonic acidemia due to coenzyme deficiency, which respond in part to therapy with B_12_ (ca. 10–20%), while the methylcobalamin deficiency blocks the methylation of homocysteine, thus causing massive homocystinuria [6]. The activity of methionine synthetase also requires the presence of some intermediates of the folate metabolism. However, congenital disorders of folate metabolism exist which, by altering the correct synthesis of these intermediates, cause a deficiency of enzymatic activity.

In particular, in 5–10, methylentetrahydrofolate reductase deficit, there is a lack of synthesis of 5 methyltetrahydrofolate which is the direct donor of the methyl group to homocysteine for the synthesis of methionine [7].

The blockade of methionine synthetase activity causes massive homocystinuria but megaloblastic anemia has never been found.

As for B_12_ there is also a folate malabsorption which is characterized by severe megaloblastic anemia with very low serum folate levels. Albeit rare there are also congenital disorders of folate metabolism, such as the functional deficit of dihydrofolate-reductase and the cellular uptake defect of folate, characterized by severe megaloblastic anemia and pancytopenia. These disorders are sensitive to therapy with folate at high doses, in case of normal serum levels of folic acid. In the few cases described it is not clear whether the pathogenesis is due to a primitive metabolic deficit of folate; in any case, aminoaciduria has never been found.

Blood cobalamin levels are not usually low because most of serum cobalamin is bound to HC and not to TC.

TC deficiency (OMIM #275350) is a rare autosomal recessive disorder: the most frequent mutations are deletions or insertions in the TCN2 gene (Cytogenetic location: 22q12.2), resulting in frame shifts that predict protein truncation [8, 9].

Currently about 60 cases of TC deficiency have been reported in the literature [10] with different presenting symptoms: from neurological (weakness, hypotonia, myoclonic like movements or delayed milestones) to psychiatric or with classic megaloblastic anemia. Other clinical manifestation of TC deficiency is gastrointestinal complications (vomiting, diarrhea, failure to thrive, and rarely oral mucositis and glossitis) [11].

Twenty-five pathogenic mutations in TCN2 gene have been identified [10]. Nonsense mutations and point mutations that activate exonic cryptic splice sites have also been reported [12, 13], as well as, polymorphic variants have also been described [8, 13].

## Case description

Our patient was born to Italian non consanguineous parents. She was born from eutocic birth with a weight of 3300 g (50th percentile), head circumference 34.5 cm (50th percentile), length 50 cm (50th percentile).

Neonatal screening for Phenylketonuria, Congenital Hypothyroidism and Cystic Fibrosis was negative. Neonatal screening for inborn errors of metabolism was not performed, because this screening in Italy was not present: it only started after a Ministerial Decree of 13 October 2016.

She was first evaluated at the age of 30 days when she presented with recurrent vomiting with every meal (exclusive breast feed), diarrhea and weight loss (< 25% percentile). Neurological evaluation was normal.

Family history revealed a sister who died at the age of 3 months with a similar clinical syndrome and with pancytopenia. This sibling was diagnosed an acute myeloid leukemia, but the diagnosis was not confirmed at post-mortem examination.

Complete blood count (CBC) revealed megaloblastic anemia (Red Blood Cell Count (*RBC)* 2,040,000/μL, Hemoglobin (Hb) 7.5 g/dL, Mean Corpuscular Volume (MCV) 115.2 fL, White Blood Cell Count (WBC) 5400/μL, platelets count 165,000/μL. Peripheral blood film showed hypochromic macrocytic RBC and hyper-segmented neutrophils, with no reticulocytosis (corrected reticulocyte count was 5%). Serum bilirubin was 7 g/dl (< 9.5).

Folate, cobalamin and total protein blood concentrations were normal (19.2 ng/mL, 1940 pg/mL and 6 g/dl respectively). Screening test for pathological amino acids revealed homocystinuria.

Urinary spot methylmalonic acid (qualitative: carried out with thin layer chromatography) [14] was not elevated, while homocystinuria was present. The study of lymphocyte subpopulations was normal (lymphocytes: T total 84%, CD3 T activated 0%, T CD4-, CD8+ 22%, T CD4+, CD8+ 0%, T CD4+, CD8–62%, Total B: DR+ 7%, B CD19 7%). Normal serum immunoglobulin levels (IgG 610 mg/dl, IgA < 6.67 mg/dl, IgM 45 mg/dl). No proteinuria was found.

Direct x-ray abdomen, digestive system after barium administration and Chest X-ray were normal.

The clinical picture, including family history, megaloblastic anemia and laboratory findings that demonstrate a normal B12 and folate level suggested the diagnostic suspect of deficiency of TC.

Therefore, treatment was started at 45 days with several transfusions of washed and concentrated RBC and intramuscular (IM) injections of hydroxocobalamin (1 mg/ day for 1 week and then 1 mg twice a week). The clinical picture progressively improved with disappearance of vomiting and diarrhea, normalization of blood tests and body growth.

At the age of 5 months, following the aforementioned treatment of IM injections of hydroxocobalamin, the infant had a normal growth: weight 6950 g (50th percentile), head circumference 45 cm (50th percentile), length 61.5 cm (50th percentile). Blood analysis: RBC Count 4,270,000/μL, WBC count 7900/μL, MCV 85 fl.

Neurological examination and Psychomotor development revealed normal findings.

In the clinical follow-up we could observe normal phases of psychomotor development, linguistic development, cognitive development and relational and socialization skills.

When she was 8 years old an attempt to suspend the treatment for about two and a half months was performed, since the parents asked to do it under clinical supervision.

This caused the appearance of pancytopenia (RBC Count 3,002,000/μL, MCV 82.5 fl, WBC 2750/μL, platelets 111,000/μL, Hb 8.9 g/dL). Therefore, IM hydroxocobalamin needed to be restarted sine die.

Menarche started at the age of 10 years and half. In the last pediatric examination at the age of 16 years, normal growth and development were observed (weight 50 kg, height 150 cm, body mass index 21.65).

Cardiovascular and neurological examinations, including Electrocardiogram (ECG) and Electroencephalogram (EEG), were normal. Dual-energy X-ray absorptiometry was within normal limits. Blood tests showed a persistent normalization of the CBC and related indices: RBC 4,570,000/μL, Hb 14.8 g/dL, MCV 88 fL,WBC 6600/μL, platelets 261,000/μL.

To clearly demonstrate the TC deficiency, we analyzed TCN2 gene to assess possible mutations. At the age of 29 years the patient underwent genetic diagnosis. The TCN2 gene (NM_000355.3) was analysed by Sanger sequencing using genomic DNA purified from patient blood. A deletion, designed c.1115_1116delCA (rs1355421014) was identified in homozygosity in exon 8. At protein level, this variation is predicted to cause the frameshift p.Q373GfsX38 resulting in a truncated protein. The mutation has a frequence of 0.000008 (1/125568, TOPMED database) or 0.0000 (0/2188, ALFA Project) and it is scored as pathogenic in ClinVar.

Patient parents screened for the mutation were found both at the heterozygous status.

At present time in 2021 the patient, who got a university degree, is a 31-years old healthy lady taking 1 mg of IM hydroxocobalamin once a week.

The timeline of events of the case report is summarized in Table 1.

**Table 1:** Timeline

Age	Symptoms	Blood Analysis	Diagnosis	Treatment	Outcome
30 days	-Pallor -Recurrent vomiting at every meal -Diarrhea -Weight loss (**<** 25th percentile)	-Megaloblastic anemia -Normal Folate and cobalamin blood concentrations -Urinary spot methylmalonic acid normal homocystinuria present	At 45 day a suspected transcobalamin 2 deficiency was diagnosed	-Several transfusions of washed and concentrated RBC and -IM injections of hydroxocobalamin (1 mg/day for one weeks and thereafter 1 mg/ twice a week)	-Vomiting and diarrhea disappearance -Blood test normalization -Body growth normalization
11 months	Any symptoms	-Normal hematological picture		IM injections of 1 mg/week of hydroxocobalamin	-Excellent health -Body weight (between the 50th and 75th percentiles)
8 years	Any symptoms	-Normal hematological picture		Therapy suspension for about two and a half months	-Pancytopenia
8 years	Any symptoms	-Pancytopenia		Therapy resumption IM injections of 1 mg/week of hydroxocobalamin	Hematological picture normalization
10 years and half	Appearence of menarche				
29 years	Any symptoms	-Normal hematological picture	Genetic confirmation of TCN2 mutation	IM injections of 1 mg/week of hydroxocobalamin	Good health
At present	Any symptoms	-Normal hematological picture		IM injections of 1 mg/week of hydroxocobalamin	She is a 31 years old healthy lady

## Discussion and conclusions

The majority of genes that could explain variation in vitamin B12 concentrations are reported from Caucasian population studies [15].

TC deficiency is a rare and potentially lethal autosomal recessive disease with an early infantile onset and the following clinical and laboratory features: failure to thrive, weakness, diarrhoea, pallor, anemia, pancytopenia, agammaglobulinemia.

This syndrome may resemble a neonatal leukemia or a severe combined immunodeficiency disease [11, 16]. Differential diagnosis with these two disease should be warranted because TC deficiency, when treated aggressively, appears to be associated with an overall favorable outcome.

TC deficiency patients usually present with megaloblastic anemia of variable severity. A delay in diagnosis and treatment are associated with life threatening neurological and hematopoietic complications, which can also be fatal [11, 17].

Patients might show isolated elevation in methylmalonic aciduria (MMA), whereas others show combined increase in circulating MMA and homocysteine [18].

In our patient there was a family history of a similar syndrome that included pancytopenia. The blood concentrations of folate and cobalamin were normal excluding these kinds of deficits, where they are low (deficiency of the absorption of B_12_, malabsorption of folate). Aminoaciduria highlighted the presence of homocysteine and the absence of methylmalonic acid, leading to the exclusion of a deficiency of methylmalonyl-CoA mutase. These findings and the presence of anemia, that is not caused by homocisteinuria alone, lead us to hypothesize the diagnostic suspicion of TC deficiency initially described in 1971 [19].

Our hypothesis was confirmed genetically only at the age of 29 years of age when we found a c.115_116delCA homozigous mutation in TCN2 gene, further confirmed by the parents’genetic analysis that showed the same mutation in the heterozygous status.

About 60 cases with TC deficiency have been reported in the literature. Twenty-five pathogenic mutations in TCN2 gene have been already identified [10], and our mutation c.115_116delCA in exon 8 was cited among these, indeed it was reported in only one patient in which this mutation was present in association with c.501_503delCCA in exon 4 [11]. In silico analysis demonstrate that exon 8 is the region involved in cobalamin binding site, whereas mutation in exon 4 should affect TC-TC receptor interaction [16].

For the first time we report the clinical significance the clinical significance of the c.115_116delCA homozigous mutation alone and not in association with other TCN2 mutations. Indeed, another case report already described is a female of 7 months who presented the same mutation of our patient (c.1115_1116 delCA) together with c.501_503delCCA [11]. Differently from our case report, she showed neurological deterioration, such as development delay, myoclonic like movements and hypotonia that were not present in our patien, who had a normal neurological development. Similarly to our case report, she diplayed pallor, thrombocytopenia and megaloblastic anemia [11]. The treatment was started at 8 months with cyanocobalamin (CN-Cbl) 1 mg IM monthly, folate p.o. switched to hydroxocobalamin (OH-Cbl) 1 mg IM 3 times a week.

She reportedly reached 32 years of age and had a normal life, but she encountered difficulties in her studies and at the age of 6, 7, and 13 years her intelligence quotient (IQ) was of 62 in Wechsler scale full. She presented progressive blurred vision and at 21 years retinopathy with partial blindness intellectual disability and depression at the age of 32 years [11] .

In our patient, the TC deficiency suspicion, before genetic screening that was performed when she was 29 years old, was indirectly confirmed by the clear response to empiric treatment with IM hydroxycobalamin once a week starting at 45 days, that provoked disappearance of vomiting, diarrhea and normalization of the hematological picture.

At present there are no guidelines on the treatment of TC deficiency regarding the form of B_12_ supplement to use (i.e. hydroxocobalamin vs cyanocobalamin), the dose, the administration method (IM vs oral), the frequency (weekly vs monthly) and the duration of administration, the treatment monitoring period and follow-up.

IM administration of 1 mg of hydroxocobalamin or cyanocobalamin once a week for a lifetime appears to be the most suitable treatment regime, according to the observational data reported in a series of 30 patients [11], that was also the treatment we found to be effective in our patient.

The working hypothesis that such a treatment regime can be correct is confirmed *ex adiuvantibus* in our patient by the observed appearance of pancytopenia, following the attempted suspension of IM hydroxycobalamin at 8 years of age. The timely resumption of treatment (3 mg in the first week, 2 mg in the second week and subsequently 1 mg per week sine die) normalized the hematological picture.

TC deficiency is a rare autosomal recessive inborn error of metabolism (prevalence: < 1/1000000), which**,** when treated aggressively**,** appears to be associated with an overall favourable outcome.

In conclusion, our case report highlights that early detection of TC deficiency and early initiation of aggressive IM with hydroxocobalamin treatment is likely associated with disease control and a favorable outcome, the temporary suspension of the therapy lead to the appearance of pancytopenia. The timely reintroduction of the treatment after about two and a half months of suspension was necessary for the renormalization of CBC. Therefore, continuing treatment sine die appears to be beneficial.

Finally, genetic counseling should be provided to affected families. Neonatal screening could be very useful in the early diagnosis of the syndrome: some studies suggest that prenatal diagnosis of TC deficiency is possible by means of measuring TC production in amniotic-fluid cells [20].

## Patient perspective

The treatment is easily administered, does not give adverse events and overall is well tolerated and safe.

## Data Availability

The data and materials generated and/or analyzed during the current study are available from the corresponding author on reasonable request.
